# Enzymatic phosphatization of fish scales—a pathway for fish fossilization

**DOI:** 10.1038/s41598-024-59025-3

**Published:** 2024-04-09

**Authors:** Fabian Gäb, Gabriele Bierbaum, Richard Wirth, Christoph Bultmann, Brianne Palmer, Kathrin Janssen, Sabina Karačić

**Affiliations:** 1 Institute of Geosciences, University of Bonn, Bonn, Germany; 2 Institute for Medical Microbiology, Immunology and Parasitology, University Hospital Bonn, Bonn, Germany; 3 Deutsches GeoForschungsZentrum (GFZ), Section 3.5 Interface Geochemistry, Potsdam, Germany; 4 Radiomed Group Practice for Radiology and Nuclear Medicine, Wiesbaden, Germany; 5 Bonn Institute of Organismic Biology, Division of Palaeontology, University of Bonn, Bonn, Germany

**Keywords:** Scanning electron microscopy, Transmission electron microscopy, Palaeontology, Microbiology

## Abstract

Phosphatized fish fossils occur in various locations worldwide. Although these fossils have been intensively studied over the past decades they remain a matter of ongoing research. The mechanism of the permineralization reaction itself remains still debated in the community. The mineralization in apatite of a whole fish requires a substantial amount of phosphate which is scarce in seawater, so the origin of the excess is unknown. Previous research has shown that alkaline phosphatase, a ubiquitous enzyme, can increase the phosphate content in vitro in a medium to the degree of saturation concerning apatite. We applied this principle to an experimental setup where fish scales were exposed to commercial bovine alkaline phosphatase. We analyzed the samples with SEM and TEM and found that apatite crystals had formed on the remaining soft tissue. A comparison of these newly formed apatite crystals with fish fossils from the Solnhofen and Santana fossil deposits showed striking similarities. Both are made up of almost identically sized and shaped nano-apatites. This suggests a common formation process: the spontaneous precipitation from an oversaturated solution. The excess activity of alkaline phosphatase could explain that effect. Therefore, our findings could provide insight into the formation of well-preserved fossils.

## Introduction

Spectacularly preserved vertebrate fossils in apatite from famous deposits have repeatedly provided breakthroughs in understanding paleobiology. Well-known examples are the southern German Jurassic Plattenkalke^1^, the Australian Devonian concretions of the Gogo-Fm^2^, the Brazilian Cretaceous Santana strata^3^, or the Eocene Green River sediments from North America^4^. In all these sites, vertebrate (fish) fossils with soft tissue preservation have been found, some with groundbreaking preservation features (e.g., reproductive organs in Gogo or muscle fibers in Santana)^2^. Although these fossils are important for understanding life history, their origin is poorly understood. Especially, the mineralization process that produced the necessary amounts of apatite remains opaque^5–10^. In this manuscript, we suggest a new pathway for the mobilization of phosphorus into an aqueous system that would enable the formation of the necessary amounts of apatite. We do not suggest that this is the only mechanism to understand these fossil deposits, however, it offers insights into the complex interplay between macro- and microbiology and inorganic, aqueous chemistry needed to form the aforementioned fossils.

### Chemical prerequisites for spontaneous apatite formation

Apatite permineralization is not a process that commonly occurs *post-mortem* in subaquatic environments. Instead, vertebrate bodies decay relatively quickly after death or are consumed by scavengers unless the decay is slowed down due to increased salinity or alkaline pH^11–14^. However, they are generally returned to the biological cycle, through decomposition, and no physiological information is retained. To achieve tissue preservation, a vertebrate body must at least be partially mineralized or imprint itself into the sediment after death instead of being degraded^15^. A transformation of the previously organic tissue into inorganic, mineral material must take place while retaining the original form of the tissue. Accordingly, pseudomorphs of the respective mineral e.g., apatite must form according to physiological structures. The chemical pathway for these mineralization processes is still poorly understood. In hard tissues such as bones, teeth, or shells, the potential for preservation is great, but the exact conditions for soft tissue preservation are still a matter of ongoing research and intensively debated, although significant progress has been made on certain mechanisms^10,11,15–18^. Here, we assessed a potential mechanism for the preservation of fish scales via apatite formation, which does not necessarily follow the same preservation processes of soft tissues.

Concerning the formation of apatite fossils, the fundamental problem is the lack of phosphorus in natural water bodies with few rare exceptions. For a fish body to completely mineralize into apatite, large amounts of phosphate must be present. However, phosphorus is a scarce resource in marine systems because it is used by algae and microorganisms for metabolism and is therefore constantly removed from seawater. The phosphorus content of seawater in the modern ocean varies between the tropical and polar regions but rarely exceeds 3 mmol/m^3^ (Copernicus Marine Service). There seems to be no critical evidence for that to have been different at least over the phanerozoic age^19^. Rather there is prominent evidence for a constant P level in the ocean throughout the phanerozoic. Recent studies suggest an even lower phosphate concentration in seawater during the Mesozoic, if compared to today, as a result of lower continental weathering at that time^20^.

This amount of phosphorus is not sufficient to replace a complete fish with apatite since the time needed to extract the phosphate from the surroundings would exceed the time the cadaver takes to decompose^12^. The body of the fish itself also does not contain enough phosphorus, since the phosphorus content of vertebrates is only ~ 1% of the body mass^21^.

However, the phosphorus content must have been increased around a fish body to form apatite and thus, form fossils. Moreover, certain environmental conditions especially the phosphorus influx from the upper sediments through vents may have played an important role. This interaction of specific inorganic conditions was already identified by Martill (1988) based on the fossils from the Santana Formation^3^. He also discussed the need for additional phosphorus sources and proposed the mentioned impact of sedimentary fluids. We want to highlight a different possibility: the enrichment of P through microbial activity. Additionally, to elevate the phosphate, the pH must have also been elevated which is a rare occurrence in any natural water bodies and is contradictive to the fact, that the decomposition of organic tissue lowers the pH^12^.

### Conditions for apatite formation

**Figure 1:**
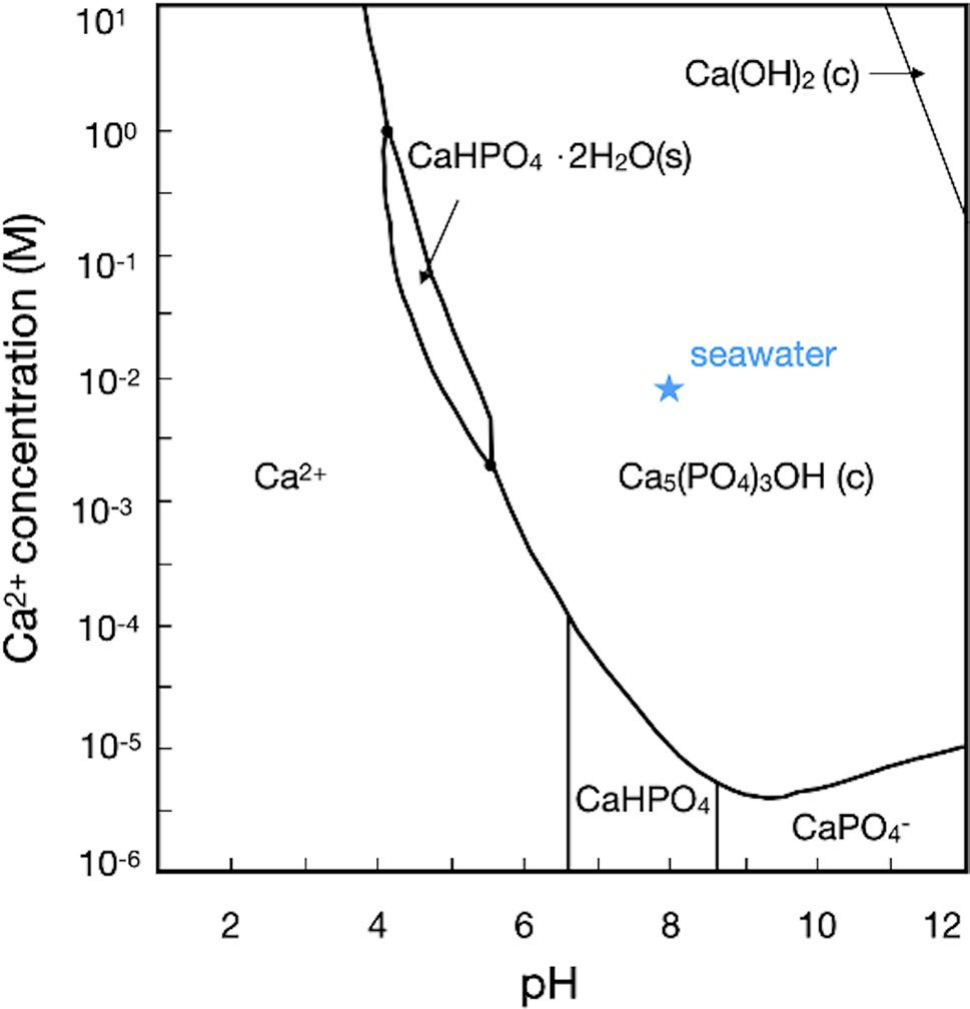
Phase diagram of calcium phosphate species in water. The position of present seawater is marked and lies within the apatite stability. (Ca concentration after^22^, pH in equilibrium with 423 ppmV CO_2_ = 2023).

Ca^2+^-phosphate concentration and pH values shown in Fig. 1 confirm that the conditions of Atlantic surface water are within the stability field of apatite. However, Gäb et al. (2020) have shown that decomposing carcasses drastically lower the pH of seawater. This effect must be buffered beforehand, otherwise, apatite would be destabilized. Furthermore, Fig. 1 and Martill (1988) show that the calcium content in seawater must be high enough for apatite to form^3^. However, seawater does contain enough calcium, which can easily be seen by the presence of calcifying organisms throughout the phanerozoic. Furthermore, a fish carcass must also stay intact long enough without decaying to become almost completely mineralized. According to literature^5,9^, biofilm formation in anoxic conditions promotes biomineralization processes by anaerobic bacteria.

Additionally, increased salinity has the same preservative effect on the fish carcass as an alkaline pH. These conditions must be pre-existent before mineralization starts. The co-occurrence of anoxic conditions and alkaline pH values is rare in most natural environments however, such conditions were described in the Black Sea^23^, thus the necessary phosphate concentration forms the major obstacle to overcome, to facilitate apatite mineralization.

### Microbes as a source of marine phosphorus

Since phosphate is a scarce resource in marine systems, marine organisms have developed methods to make phosphate available^24^. Like most other organisms, marine microorganisms synthesize the enzyme alkaline phosphatase (AP). This enzyme cleaves phosphate from phosphoric acid esters in which it is fixed, mostly from easy degradable sources like ADP or ATP. This makes bound phosphate reusable for organisms. Because they live in a phosphate-deficient environment with an irregular influx of phosphorous from concentrated organic phosphate sources (e.g. dead algae, dead fishes), many marine bacteria and algae can produce putative AP-variants^25^. In combination with other exoenzymes and DNase, they are also capable of breaking down recalcitrant organic phosphates like DNA or phosphorylated amino acids released by decaying organisms or decaying microbial or algal blooms into the environment^26^. This mechanism is used for the recuperation of phosphorus from the sewage at the biological clarification stage in sewage treatment plants^22^. In this setting, phosphate oversaturation and precipitation of inorganic hydroxylapatite (HAP) can be observed regularly^27^.

Through this process, on a larger scale in lakes or the open sea, bacteria can influence the phosphate content of the surrounding water^28^. The activity of AP may increase the inorganic phosphate content in seawater to HAP saturation and inorganic HAP precipitates from seawater^29^. This is considered a potential source for the formation of marine phosphate deposits in North Africa^30^. Thus, it is also conceivable that a fish carcass could be apatized by higher AP activity increasing the phosphate content of seawater in its vicinity to the point that inorganic HAP is precipitated. As shown in the fossil record, near-complete mineralization in apatite of fish has occurred several times in Earth’s history^1–4^. However, various studies highlight the importance of the role of microbes and their activities in soft tissue preservation^15,31^. Thus, microbes could also be important in organic tissue preservation such as fish scales.

In this paper, we test if the activity of the enzyme alkaline phosphatase could lead to the phosphatization of organic tissue. We propose that the overactivity of the enzyme alkaline phosphatase (AP) produced by microbes is a possible method for the origin of phosphatized fish. To test the hypothesis that AP activity can lead to apatite permineralization of fish, we performed experiments, analyzed samples with scanning electron microscope (SEM) and transmission electron microscopy (TEM) and compared the results to natural fossils, also analyzed via SEM and TEM. Since this is ultimately an inorganic precipitation in which only the necessary concentrations were produced by biological processes, the products can be compared with those of other inorganic precipitation processes in seawater.

## Methods

### Phosphatization experiments

All experiments listed in Supplementary Table 1 were performed in the S2 laboratory of the Institute of Medical Microbiology, Immunology, and Parasitology at the University Hospital Bonn. Fresh dorades (*Sparus aurata*) were obtained from local groceries. The fish scales of the dorades served as a substrate for apatite formation in each experiment. The fish scales were autoclaved and stored in sterile H_2_O MilliQ^®^ in 50 ml falcon tubes at 4 °C. The bacterial cultures (*E. coli* JM109 and *M. luteus* ATCC4698 that served as sources of phosphate were prepared by inoculating 25 ml of Trypticase Soy Broth (TSB) medium with the relevant strain and incubating at 37 °C overnight. The cultures were then centrifuged at 5000 rpm at 4 °C for 15 min. Afterwards, the supernatant was discarded, and the pellet was resuspended in 700 μl sterile 20 mM Tris buffer (pH 9–10, containing 200 mM CaCl_2_) (Supplementary Table 1). Since AP has maximum effectiveness in the alkaline range (at pH ~ 9)^32^, the buffer was also adjusted to this pH, and the bacterial culture was transferred into Eppendorf tubes and centrifuged at 12.000 rpm at 4 °C for 2 min twice.

After each centrifugation step, the supernatant was discarded, and the pellet was resuspended in 1 ml Tris buffer with 200 mM CaCl_2_. Finally, the OD_600_ was measured, and the concentration was adjusted to OD 1. To ensure effective access to the phosphate source, lysozyme (10 mg/ml) was used for bacterial cell lysis and DNase and RNase from bovine pancreas (1 mg/ml, Roche Diagnostics GmBH) for breakdown of bacterial DNA and RNA.

Supplementary Table 1 provides an overview of experimental approaches, composition, and the respective volumes. The formation of apatite was induced by the addition of commercial AP (bovine AP 10–20 mg/ml, Carl Roth®). The muscle tissue of dorade fish, 4-nitrophenyl phosphate (NPP 0.6 mM), and bacterial biomass (OD 0.1) were alternately added to the experiment as a substrate for the enzyme AP. NPP is a synthetic organic compound used to detect alkaline phosphatase activity. It is therefore used as positive control. After preparation of the fish scale samples, they were incubated at 25 °C, 37 °C, or 4 °C for 1, 2, or 3 weeks before termination by pasteurization. After this process, the samples were 10 min heated to 60 °C, to denature the enzyme. Samples from completed experiments were analyzed via SEM with EDS (energy-dispersive X-ray spectrometry) to identify conditions that produced apatite crystals. Afterwards, the most prominent apatites were analyzed by TEM to characterize the crystallographic properties and the nano-structure of the precipitates.

A TESCAN Vega 4 SEM coupled with EDS (Institute of Geosciences, University of Bonn), was used to analyze the experiments. The fish scales were removed from the solution, air-dried, and then glued to aluminum stubs with conductive carbon plates and sputter-coated with carbon using a Cressington Coater. SEM analysis was performed at an accelerating voltage of 20 keV in high vacuum mode. During SEM analysis the aim was to identify possible apatite formation. This could easily be seen since the newly formed apatite grains lay on top of the fish scale. To verify the composition of possible apatite finds, they were characterized by EDS.

### Investigation of fossils

In addition to analyzing the results of these experiments, we also analyzed a fossil fish from the genus *Gyrodus* (Mönsheimer Fm, Altmültal, S’ Germany) from the upper Jurassic (Thithonian ~ 152 Ma) with SEM and TEM to identify similarities between the experimental results and existing fossils. The fossil was cut into cubes with a side length of ~ 1 cm and stabilized in epoxy. After SEM analysis the sample was analyzed with TEM at the GFZ Potsdam along with a detailed analysis of the apatite grains that were produced in the experiments. Both, fossil material and experimental apatite, were analyzed with TEM.

Based on our previous results, we checked another apatized fossil fish, of the genus *Rhacolepsis* (Santana Fm. Brazil) from the upper Cretaceous (~ 80 Ma). We prepared the fossil in the same way as the previous fossil and observed it under TEM.

### Transmission electron microscopy

Electron transparent foils for TEM studies were prepared with a focused ion beam (FIB system type HELIOS. The thin foils were sputtered out of the target material using Ga-ions. The rough sputtering occurred at 30 keV with a subsequent polishing of the sample at 5 keV. The dimensions of the foils are approximately 15 μm × 10 μm × 0.1 μm. Details of FIB sample preparation are presented elsewhere (Wirth, 2009).

TEM was performed with a TECNAI F20 Y-Twin operated at 200 keV with a Schottky field emitter as an electron source. The TEM is equipped with a Gatan energy filter Tridiem, a Fischione high-angle annular dark field (HAADF) system and an EDAX X-ray analyzer. TEM images were acquired as energy-filtered images applying a 20 eV window to the zero-loss electron beam. EDS analyses were acquired in the scanning transmission mode (STEM) scanning the electron beam across a preselected area, thus avoiding mass loss by electron sputtering. Acquisition time usually was 60 s.

## Results

The experiments combining fish scales and commercial AP succeeded in verifying the basic approach that AP can be used to achieve a local phosphate concentration that leads to the inorganic precipitation of apatite. The efficiency of the process depends on the availability and suitability of the substrate offered. The results ranged from precipitation of isolated, small (~ 5 µm) crystals of apatite, to larger individual crystals (~ 100 µm), to complete coverage of entire fish scales with a layer of newly formed apatite. The distinction between apatite primarily present in a fish scale and newly formed apatite is recognizable by the crystal shape and given by an analysis of the P content using an EDS map.

The first series of experiments used NPP as a substrate. Since NPP was designed to verify the activity of AP, it was easily broken down by AP and provided excess phosphate (see Eq. 1). NPP was thus the ideal substrate for AP and the resulting phosphate concentration in the solution, and thus precipitation, was maximal.
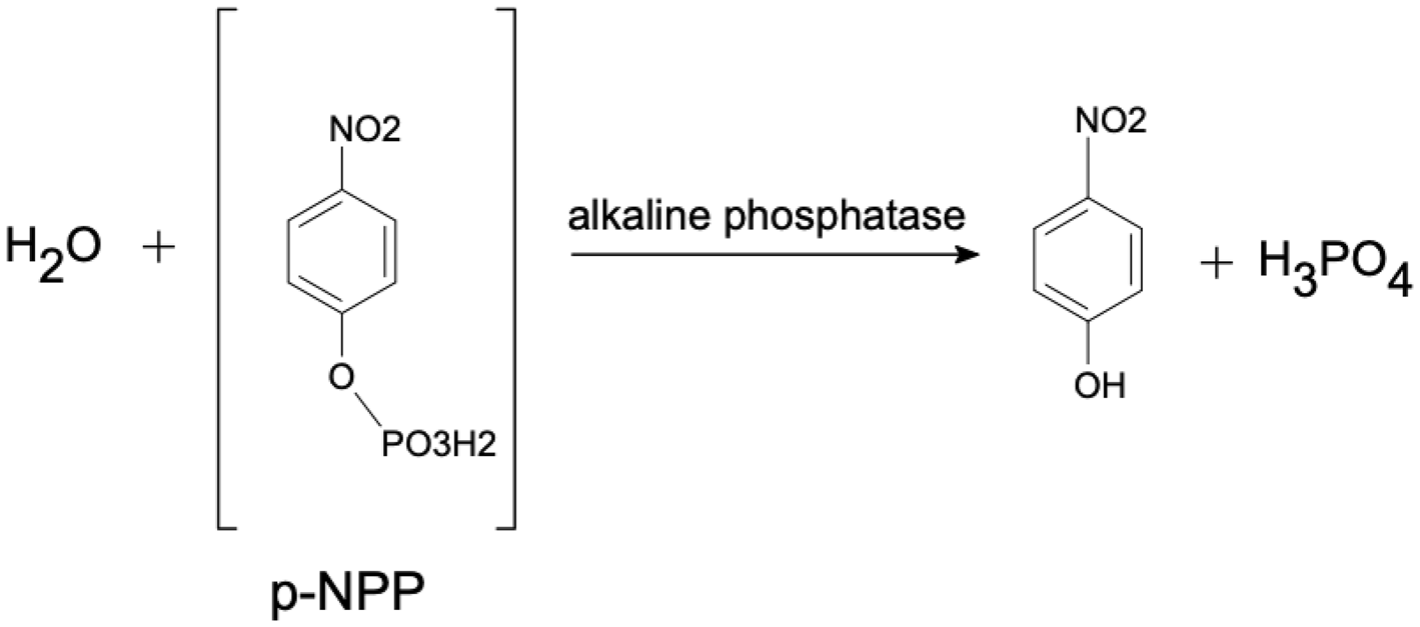


**Figure 2:**
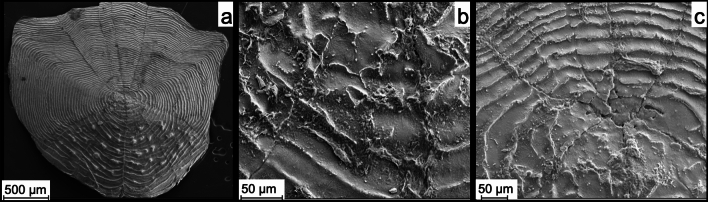
SEM image (SE) of experiment FAV 1_e. A fish scale with 50 µl AP, and 100 mM NPP in seawater buffer for 1 week. (**a**) shows the whole scale, where the growth rings can be seen. (**b**) shows a detailed view and that the scale is covered with newly formed apatite. (**c**) shows that the newly formed apatite replicates the primary physiological information (growth rings). Note that the whole scale is covered in newly formed apatite and the original surface cannot be seen anymore.

The complete fish scale reacted with 50 µl AP and 100 mM NPP for one week in a seawater buffer (Fig. 2, experiment FAV 1_e Supplementary Table 1). Here the primary physiological information (the growth rings of the scale) remained recognizable and new crystals precipitated (Fig. 2b). Between the individual growth rings there was precipitated apatite, in addition to the apatite-cover.

This illustrates the degree of supersaturation that occurred in the experiment. Furthermore, the formation of these crystals indicated ongoing activity of the AP, since the crystals were located on the layer that precipitated first and subsequently additional crystals were formed on top of the primary layer of apatite. Thus, the enrichment of the solution with phosphate continued even after the initial precipitation of apatite. However, there was an excess of both NPP and AP in this experiment.

The level of detail of the apatite coverage leaves the physiological structures visible (Fig. 2c). This is a prerequisite for fossilization, as the preservation of physiological information is essential. This result showed that supersaturation of a solution to inorganic precipitation of calcium phosphate (apatite) was possible by the activity of AP. The breakdown of NPP by AP shown in Eq. (1) resulted in a large increase in phosphate concentration, therefore, the degree of oversaturation for apatite (Ωap) increased resulting in spontaneous crystallization. This precipitation then replicated primary organic structures. An organic structure covered in this form of newly formed apatite may have fossilization potential because the essential features were templated in apatite. However, this was not a permineralization sensu strictu, but rather a mineral cover on organic material.

The approach to use NPP as a substrate led to the verification of our basic hypothesis: AP activity can increase the phosphate content to concentrations where apatite is precipitated inorganically. The next set of experiments used a more naturalistic approach using natural substrates. Using muscle tissue from the fin ray of a dorade as a substrate did not provide any insights, nor did the use of *E. coli* bacteria in the absence of lysozyme, and these substrates were therefore discarded (Supplementary Table 1). There was no improvement in the presence of DNase or RNase. The next set of experiments used *M. luteus* pellets and lysozyme in solution, to study the effect of AP systematically. These experiments resulted in detectable crystals on the fish scales.

### SEM

**Figure 3:**
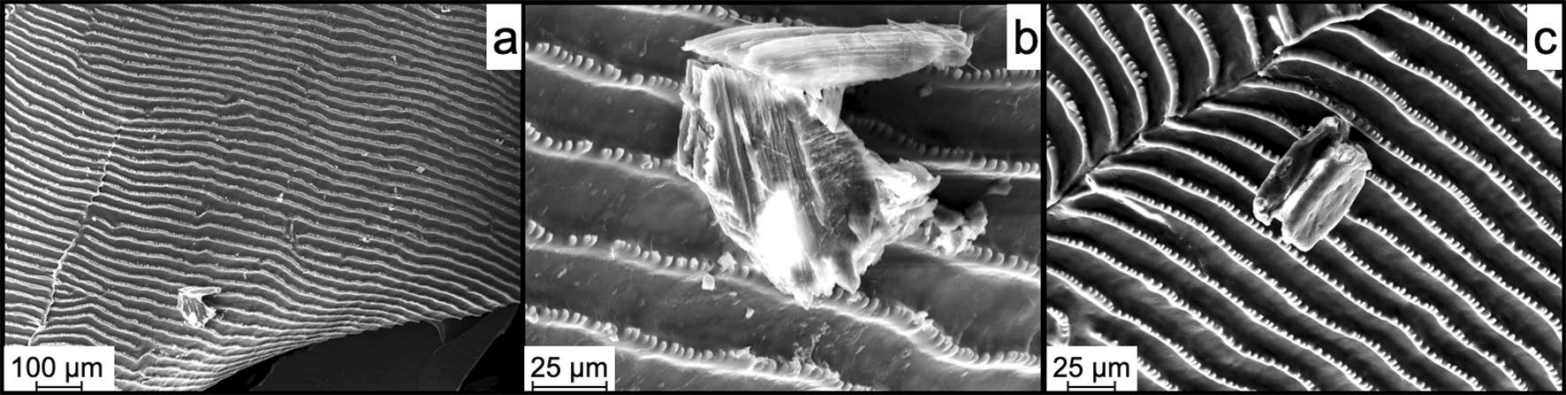
SEM images (SE) of different experiments where small apatite crystals were precipitated. These experiments were performed using pasteurized bacterial biomass as substrate (see “Methods”). The crystals appeared as small apatite grains (size ~ 50 µm) on top of the target material (fish scale) as can be seen in (**a**) and had an angular shape with clear crystal surfaces (**b**). The composition was verified by EDS. (**c**) Different grain from a replicate experiment.

We analyzed the samples with SEM to confirm the precipitation of potential apatite on the samples. Apatite grains were readily identified by SE imaging in combination with EDX. The newly formed crystals were distinguishable from the target material (fish scale) (Fig. 3) and possessed an angular shape. In experiments that did produce apatite there was usually more than just one apatite, all with similar same size and shape. However, the grain shown in Fig. 3c was smaller and less angular. These results were reproduced several times, showing that the solution in these experiments became oversaturated with calcium phosphate.

The first experiments with the setup containing *M. luteus* bacteria, lysozyme, and bovine AP only produced a few small apatite grains. Replicates of these experiments did produce larger apatite crystals.

**Figure 4:**
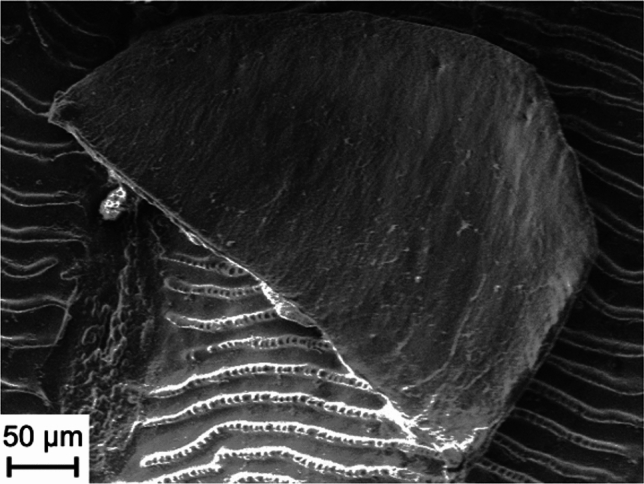
SEM image (SE) of a larger (~ 800 µm) apatite. Please note: The apatite appears to be broken in half and could have been even larger before breaking. Its shape resembled the curvature of the fish scale. The composition was verified by EDS.

We observed a large apatite (Fig. 4) on the fish scale sample from the experimental setup with the same composition (bovine AP, *M. luteus* as substrate, lysozyme, and TRIS buffer). The reason for the difference in the size of the generated grain, and perhaps improving the efficiency of the phosphate liberation reaction, is currently unknown. The shape of this apatite resembles the curvature of the fish scale lying underneath. Additionally, it looked like the grain had been broken in the middle. This suggests that the original object was even larger before and potentially covering the fish scale.

**Figure 5:**
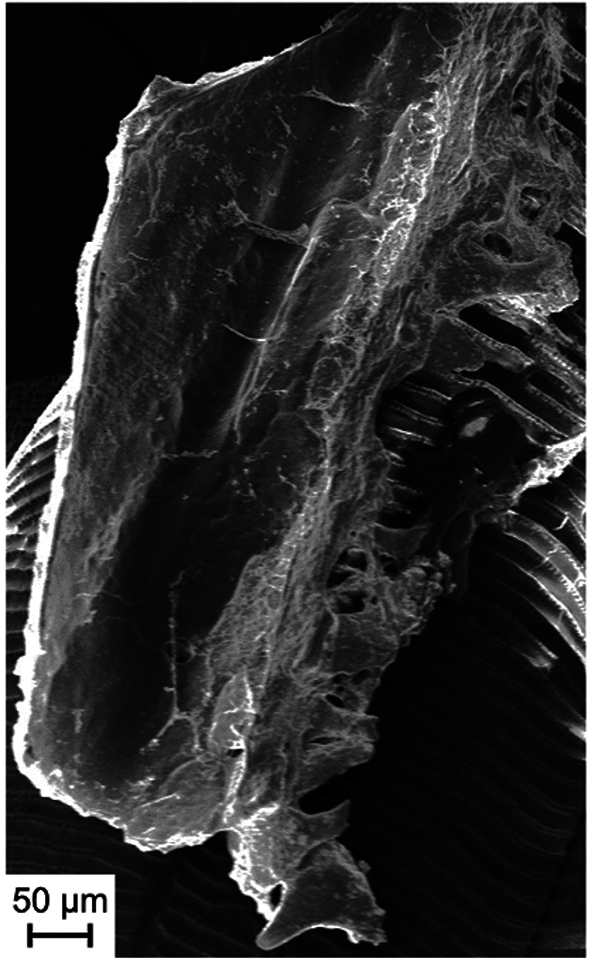
SEM image of the largest (~ 1 mm) apatite found in the experiments. This grain resulted from an experiment with the same composition as described above (bovine AP, *M. luteus* as substrate, lysozyme, and TRIS buffer). This grain was later analyzed by TEM.

The largest apatite produced in these experiments had a grain size of ~ 1 mm (Fig. 5). As with the grain shown in Fig. 4, the apatite showed signs of damage possibly due to transport compared to its original shape. We can also assume that this grain was larger at the point of formation. Because of its size, this apatite was used for further analysis with the TEM. Following the experiments with apatite sizes ranging from 50 µm to 1 mm, we were unable to identify which set of parameters would lead to a guaranteed success of the experiment.

### TEM

**Figure 6:**
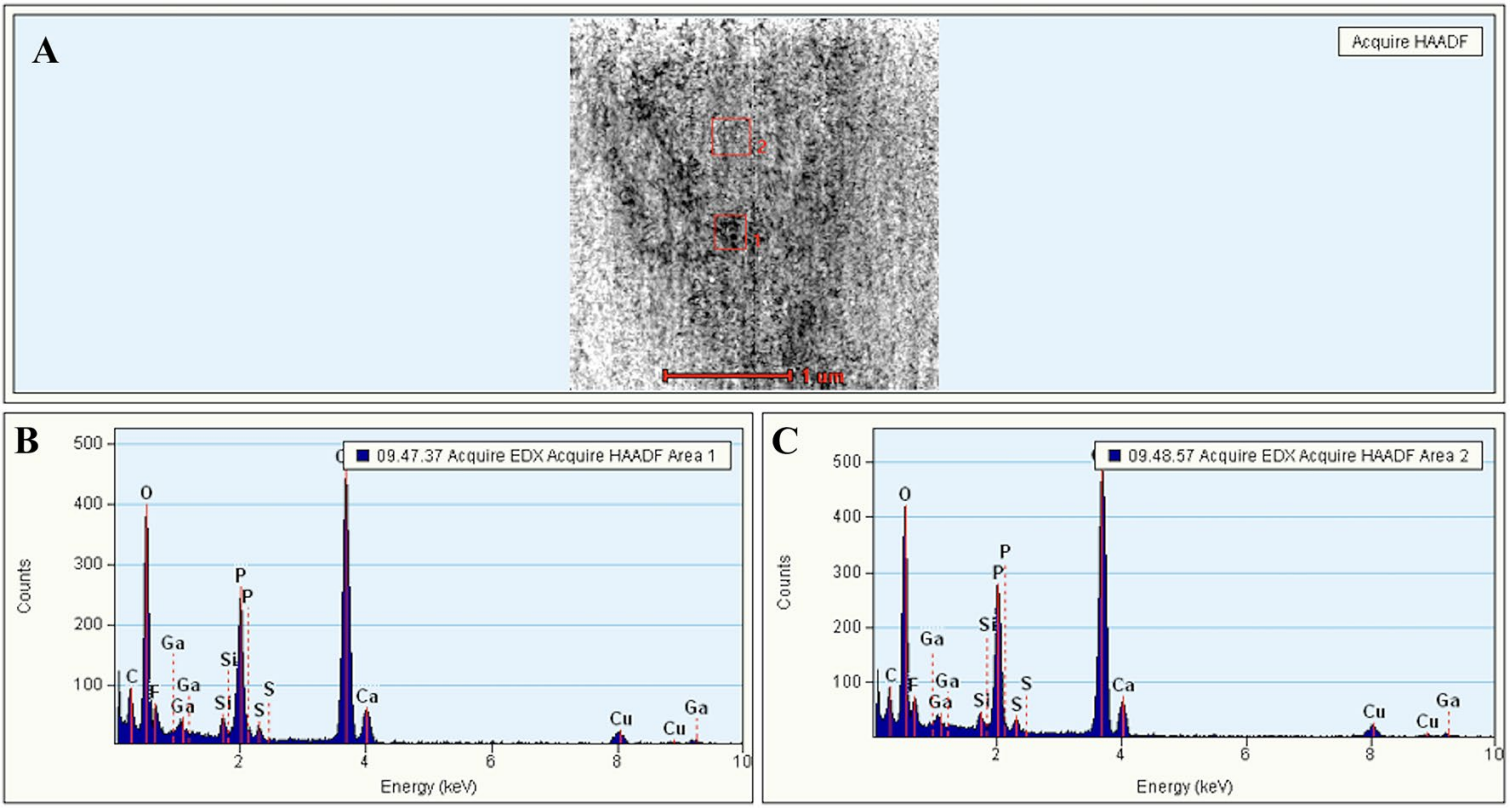
EDX analyses of the *Gyrodus* sample. Calcium, phosphorus, and oxygen are typical for apatite. Fluorine is typical in natural apatites from sediments. (**A**) Overview. Two marked sections of the fossils were examined in (**B**) and in (**C**).

The EDX analyses showed a uniform picture of the mineralogy of the fossils in all examined sections. The fossils consist entirely of (fluoro)-apatite crystals with only low contents of silicon and sulfur, which was verified via EDS. The results of these analyses are shown in Fig. 6.

The high carbon content observed in the samples was the result of sputter coating with carbon before SEM analysis as well as from the treatment with epoxy resin. Furthermore, the sample contained calcite which was verified with EDS. The low gallium contents result from the focused ion beam (FIB) sputtering and Ga-ion implantation. Additionally, small amounts of fluorine were detected in the sample, as can be expected in apatites of this age, as most apatites accumulate fluorine and carbonate from the environment during diagenesis (francolite-formation). The composition of the apatites does not vary within the measured samples, nor does it vary between the different cuts through the fossil.

#### TEM of the fossil

The high resolution of the TEM allowed for visualization of individual apatite crystals in the fish fossils. Bright-field analysis of the samples showed apatite nanocrystals of comparable habit and size in all examined areas of the fossil. They were about 40 nm long and about 15 nm wide with little variation in crystal size. Crystals with a size of > 150 nm were not detected.

**Figure 7:**
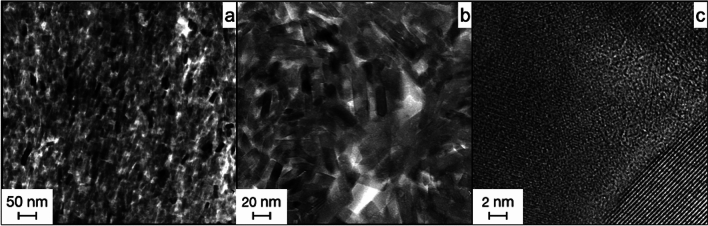
Results from the TEM analysis of the fossil apatite from *Gyrodus* in Solnhofen. (**a**,**b**) show bright-field images of the fish fossil. The apatite here is composed of ~ 20 nm large single crystals. Dark contrast crystals are those with a low-indexed zone axis oriented parallel to the electron beam. HREM (high-resolution TEM) is used for (**c**) to show the amorphous pore space between the individual nanocrystals with lattice fringes visible thus indicating crystallinity.

The habit of the nanocrystals corresponds to the idiomorphic crystal form of hydroxyapatite; the longitudinal sections of tabular prisms can be seen in Fig. 7a. They correspond to plane of a hydroxyapatite crystal. In Fig. 7a, the crystals look oriented, but this does not correspond to the overall appearance of the specimen. In general, the crystals were arranged at random and were not aligned. We observed the primary porosity between the grains. The crystals had precipitated chaotically in three dimensions.

Moreover, dark head cuts of the prisms were observed. Furthermore, pores between the individual crystals, in which no crystalline material was present, were visible. These interstices can be classified more precisely through HREM (Fig. 7c). Here, the interstices between the individual apatite nanocrystals were amorphous and did not show a clear crystal lattice. The crystals can easily be identified in the bottom right corner. The crystal lattice was clearly defined by lattice fringes.

In summary, the fossil material was composed of apatite nanocrystals of approximately 20 nm in size. They were not ordered and there was an amorphous rim around each crystal isolating it from direct contact with the next crystal. TEM of the *Rhacolepsis* fossil from Santana Fm. showed an almost identical structure of the apatite. The fossil here was also made up of apatite nanocrystals and had the same properties (e.g., size, shape, chaotic arrangement, and amorphous pore space).

#### TEM of the experimental apatite

TEM of the experimentally generated apatite did show a nearly identical image as TEM of the fossil apatite.

**Figure 8:**
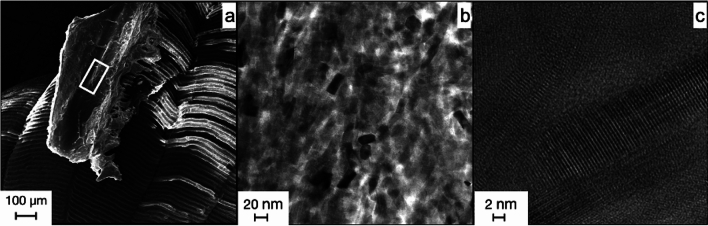
Results from the TEM analysis of the experimental apatite. (**a**) shows the position the sample was taken from the grain of apatite. (**b**) shows a bright-field image of the nanocrystals that make up the larger grain. Crystals with dark contrast are oriented with a low-indexed zone axis in parallel to the electron beam. (**c**) shows the HREM results and demonstrates that the crystals in the experimental apatites are separated by amorphous material from each other.

The experimentally generated apatite in Fig. 8 is the same experimental apatite that was described in Fig. 5. Interestingly, the chaotic layering, size, and overall appearance are almost identical to the detailed images taken from the fossil material (c.f. Figure 7). Moreover, the results of the HREM analysis (Fig. 8c) resemble the fossil material. The individual nanocrystals are readily identified by a clear crystal lattice as lattice fringes, whereas the pore space in the crystal interstices does not show lattice fringes and is thus, amorphous.

The striking resemblance between the 150 Ma old fossil material and the freshly generated apatite from the experiment is easily recognizable. Both contained apatite with similar crystallographic properties. This could mean that these two materials were both precipitated from an oversaturated solution.

## Discussion

In this study, in vitro experiments were used to investigate a natural process. Employing commercial AP, it was possible to precipitate apatite in the laboratory which strongly resembled in its structure the apatite that was found in fossils. A total coverage of the fish scales was obtained when providing the commercial substrate for AP, however, only single crystals were detected using bacterial biomass as a substrate.

The results presented here are intended as a proof of concept. However, the experiments provide evidence for the formation of fossils in the above-mentioned deposits. We showed that, in principle, it is possible to modify the phosphate content in seawater by enzymatic activity in such a way that inorganic precipitation of apatite occurs. This is not trivial information, because the fundamental difficulty in considering phosphatized fossils is always the contradiction between a phosphate precipitation, i.e., an excess, and a fundamental phosphate deficiency in natural seawater. Locally, however, this contradiction can be resolved by AP activity. The degree of supersaturation, which is correlated with the amount of precipitated apatite, is in turn dependent on the concentration of AP and the quality of the substrate provided. For an ideal substrate such as NPP, the amount of precipitated apatite is very large. In the experiments where the bacterial biomass was not previously prepared by lysozyme, i.e., the substrate was very complex, little to no apatite was precipitated. Moreover, there is a competitive situation between apatite and calcite. Both minerals are stabilized at alkaline pH values and are then able to precipitate inorganically from a solution^12^. In addition, since both minerals incorporate Ca^2+^, the precipitation of one mineral is an inhibitor of the precipitation of the other.

Since these apatite precipitates are only coverings and not permineralization of primarily organic material, these results raise questions about the applicability of certain methods, especially in isotope geochemistry on fossils. If phosphorus is injected into the system from external sources, the oxygen isotopy might be altered post-mortem. Furthermore, the phosphate formed here would not be in equilibrium with any phosphorus present in the body. Thus, mass balancing of these reactions would not provide any further insight.

Despite the success of these experiments, a limitation of this study is the use of bovine AP. Due to its mammalian origin, bovine AP is not a realistic candidate for the actual process of fossilization, which has been happening throughout Earth’s history, although it does occur in recent seawater in lower abundance^33^. Bovine AP belongs to the PhoA family of alkaline phosphatases. These enzymes are found in higher animals, plants, and bacteria^34^ and are product-inhibited, so the activity of the enzyme decreases if the amount of the product, here phosphate ions, increases^35^. Moreover, the substrate range of bovine PhoA is limited to phosphomonoesters, pyrophosphate and the 5ʹtermini of DNA or RNA molecules^36^. The PhoX family phosphatases represent more realistic candidates for the release of phosphate in nature, however, these enzymes are not commercially available. PhoX APs are ubiquitous in marine microbes^28,33,37–39^ secreted into the water^40^, operate in a wide range of environmental conditions^41^ and are less restricted to a specific substrate^33^. Future studies will test PhoX in similar experiments.

The other limitation in our experimental setup is the use of a complex substrate in the form of a bacterial cell pellet of *M. luteus*, which was chosen to represent decaying biomass. The bacterial cell walls are destroyed by the added lysozyme and the protoplasts burst, releasing their contents. In some experiments, DNase was added to release nucleotides. However, DNAase is more effective at lower pH than which was present in the experimental conditions. In addition, membrane lipids with their long hydrophobic tails are not an ideal substrate for bovine PhoA because the enzyme usually works on smaller molecules^34^. Moreover, we used autoclaved fish scales to avoid microbial biofilm formation on scales. The autoclaving pre-treatment likely changed the scale and effect of crystal nucleation. Therefore, the efficiency of the phosphate generation from this rather complex substrate may be low. This was demonstrated with the apparent high efficiency of the precipitation reaction when using NPP as substrate as we did in the first experiments. Here, if provided with an ideal substrate, the setup showed the large amounts of apatite that can be produced by the activity of AP.

Despite all limitations regarding the complexity of the experimental system, these findings hold significant value when compared to natural sites. The processes of apatite precipitation induced by phosphate enrichment via microbial activity, shown in our experimental work, occur regularly in sewer plants^27^ on a large scale and almost naturalistic environment. The technical setups take into consideration the complexity, size, pH, and Eh-values and microbial population surrogates for our proposed fossilization hot spots.

Given that together with the findings in this study, there are laboratory and technical occurrences of the shown process, it is likely to assume that under certain conditions a similar process could happen in a 100% natural system and result in apatized fossils.

## Conclusion

We showed that it is indeed possible to increase the amount of dissolved phosphate in a solution to the degree of apatite solution via alkaline phosphatase activity. Even with several disadvantages concerning efficiency e.g., bovine AP, insufficiently processed substrate, or less than optimal pH, apatite has been produced consistently throughout the experimental series. This shows the potential of the method for fossilization processes. The TEM results indicate that fossil material and apatite generated by our experiment share many crystallographical properties, which further strengthen the hypothesis of both sharing a common way of formation. Although these results are more a proof of concept, they do, in our opinion, validate further research.

### Supplementary Information

Supplementary Table 1.

## Data Availability

The datasets used and/or analysed during the current study are available from the corresponding author on reasonable request.
