# Supplementary material for: Enzymatic phosphatization of fish scales—a pathway for fish fossilization
Source: Sci Rep. 2024 Apr 9;14:8347. doi: 10.1038/s41598-024-59025-3 (PMC11003971; doi:10.1038/s41598-024-59025-3)
Supplement: Supplementary file 1 — Supplementary Table 1. [file 41598_2024_59025_MOESM1_ESM.docx]

**Supplementary material**

Table 1: Experimental Overview.

| sampleID | control | target | subs. | AP  [µl] | lysosyme | buffer | temp. | duration | DNAase | RNAase |
| --- | --- | --- | --- | --- | --- | --- | --- | --- | --- | --- |
| FAV 1_a | n | Fish scales | *E. coli* | 10 | n | seawater | 25°C | 1 week | n | n |
| FAV 1_b | n | Fish scales | *E.coli* | 10 | n | seawater | 25°C | 1 week | n | n |
| FAV 1_c | y | Fish scale | n/a | 10 | n | seawater | 25°C | 1 week | n | n |
| FAV 1_d | n | Fish scale | NPP | 10 | n | seawater | 25°C | 1 week | n | n |
| FAV 1_e | n | Fish scale | NPP | 50 | n | seawater | 25°C | 1 week | n | n |
| FAV 1_f | n | Fish scale | NPP | 1 | n | seawater | 25°C | 1 week | n | n |
| FAV 1_g | y | Fish scale | n/a | 0 | n | seawater | 25°C | 1 week | n | n |
| FAV 2_C1 | y | Fish scale | *E.coli* | 0 | n | TRIS pH 9,3 | 25°C | 2 weeks | n | n |
| FAV 2_C2 | y | Fish scale | *E.coli* | 0 | y | TRIS pH 9,3 | 25°C | 2 weeks | n | n |
| FAV 2_1 | n | Fish scale | *E.coli* | 2 | n | TRIS pH 9,3 | 25°C | 1 week | n | n |
| FAV 2_2 | n | Fish scale | *E.coli* | 2 | y | TRIS pH 9,3 | 25°C | 1 week | n | n |
| FAV 2_3 | n | Fish scale | *E.coli* | 2 | n | TRIS pH 9,3 | 25°C | 2 weeks | n | n |
| FAV 2_4 | n | Fish scale | *E.coli* | 2 | y | TRIS pH 9,3 | 25°C | 2 weeks | n | n |
| FAV 3_C1 | y | Fish scale | n/a | 2 | n | TRIS pH 9,3 | 37°C | 2 weeks | n | n |
| FAV 3_C2 | y | Fish scale | n/a | 2 | y | TRIS pH 9,5 | 37°C | 2 weeks | n | n |
| FAV 3_C3 | y | Fish scale | NPP | 2 | n | TRIS pH 9,3 | 37°C | 2 weeks | n | n |
| FAV 3_C4 | y | Fish scale | NPP | 2 | y | TRIS pH 9,5 | 37°C | 2 weeks | n | n |
| FAV 3_1 | n | Fish scale | *M.luteus* | 2 | n | TRIS pH 9,3 | 37°C | 1 week | n | n |
| FAV 3_2 | n | Fish scale | *M.luteus* | 2 | y | TRIS pH 9,5 | 37°C | 1 week | n | n |
| FAV 3_3 | n | Fish scale | *M.luteus* | 2 | n | TRIS pH 9,3 | 37°C | 3 weeks | n | n |
| FAV 3_4 | n | Fish scale | *M.luteus* | 2 | y | TRIS pH 9,5 | 37°C | 3 weeks | n | n |
| FAV 3_5 | n | Fish scale | *M.luteus* | 2 | n | TRIS pH 9,3 | 37°C | 1 week | n | n |
| FAV 3_6 | n | Fish scale | *M.luteus* | 2 | y | TRIS pH 9,5 | 37°C | 1 week | n | n |
| FAV 3_7 | n | Fish scale | *M.luteus* | 2 | n | TRIS pH 9,3 | 37°C | 3 weeks | n | n |
| FAV 3_8 | n | Fish scale | *M.luteus* | 2 | y | TRIS pH 9,5 | 37°C | 3 weeks | n | n |
| FAV 3_CF2 | y | Fish scale | n/a | 2 | y | TRIS pH 9,5 | 4°C | 6 weeks | n | n |
| FAV 3_CF3 | y | Fish scale | NPP | 2 | n | TRIS pH 9,5 | 4°C | 6 weeks | n | n |
| FAV 3_F1 | n | Fish scale | *M.luteus* | 2 | n | TRIS pH 9,5 | 4°C | 6 weeks | n | n |
| FAV 3_F2 | n | Fish scale | *M.luteus* | 2 | y | TRIS pH 9,5 | 4°C | 6 weeks | n | n |
| FAV 4_C1 | y | Fish scale | n/a | 2 | y | TRIS pH 9,3 | 37°C | 1 week | n | n |
| FAV 4_C3 | y | Fish scale | *M.luteus* | 2 | y | TRIS pH 9,3 | 37°C | 1 week | n | n |
| FAV 4_C4 | y | Fish scale | *M.luteus* | 2 | y | TRIS pH 9,3 | 37°C | 1 week | n | n |
| FAV 4_1 | n | Fish scale | *M.luteus* | 2 | y | TRIS pH 9,5 | 37°C | 1 week | n | n |
| FAV 4_2 | n | Fish scale | *M.luteus* | 20 | y | TRIS pH 9,3 | 37°C | 1 week | n | n |
| FAV 4_3 | n | Fish scale | *M.luteus* | 20 | y | TRIS pH 9,5 | 37°C | 1 week | n | n |
| FAV 5 FS1 | n | Fish scale | *M.luteus* | 2 | y | TRIS pH 9,5 | 37°C | 2 weeks | n | n |
| FAV 5 FS2 | n | Fish scale | *M.luteus* | 2 | y | TRIS pH 9,3 | 37°C | 2 weeks | n | n |
| FAV 5 FS3 | n | Fish scale | *M.luteus* | 2 | y | TRIS pH 9,3 | 37°C | 2 weeks | n | n |
| FAV 5 FS4 | n | Fish scale | *M.luteus* | 20 | y | TRIS pH 9,3 | 37°C | 2 weeks | n | n |
| FAV 5 FS5 | n | Fish scale | *M.luteus* | 20 | y | TRIS pH 9,3 | 37°C | 2 weeks | n | n |
| FAV 5 FS6 | n | Fish scale | *M.luteus* | 20 | y | TRIS pH 9,3 | 37°C | 2 weeks | n | n |
| FAV 6 FS1 | n | Fish scale | *M.luteus* | 2 | y | TRIS pH 9,3 | 37°C | 4 weeks | n | n |
| FAV 6 FS2 | n | Fish scale | *M.luteus* | 2 | y | TRIS pH 9,3 | 37°C | 4 weeks | n | n |
| FAV 6 FS3 | n | Fish scale | *M.luteus* | 2 | y | TRIS pH 9,5 | 37°C | 4 weeks | n | n |
| FAV 6 FS4 | n | Fish scale | *M.luteus* | 20 | y | TRIS pH 9,5 | 37°C | 4 weeks | n | n |
| FAV 6 FS5 | n | Fish scale | *M.luteus* | 20 | y | TRIS pH 9,3 | 37°C | 4 weeks | n | n |
| FAV 6 FS6 | n | Fish scale | *M.luteus* | 20 | y | TRIS pH 9,3 | 37°C | 4 weeks | n | n |
| FAV 7 D1 | n | Fish scale | *M.luteus* | 5 | y | TRIS pH 9,5 | 37°C | 2 weeks | n | n |
| FAV 7 D2 | n | Fish scale | *M.luteus* | 5 | y | TRIS pH 9,5 | 37°C | 2 weeks | n | n |
| FAV 7 E1 | n | Fish scale | *M.luteus* | 5 | y | TRIS pH 9,5 | 37°C | 2 weeks | y | n |
| FAV 7 E2 | n | Fish scale | *M.luteus* | 5 | y | TRIS pH 9,5 | 37°C | 2 weeks | y | n |
| FAV 7 F1 | n | Fish scale | *M.luteus* | 5 | y | TRIS pH 9,3 | 37°C | 2 weeks | y | y |
| FAV 7 F2 | n | Fish scale | *M.luteus* | 5 | y | TRIS pH 9,3 | 37°C | 2 weeks | y | y |
| FAV 7 1 | n | Fish scale | *M.luteus* | 5 | y | TRIS pH 9,3 | 37°C | 2 weeks | n | n |
| FAV 7 2 | n | Fish scale | *M.luteus* | 5 | y | TRIS pH 9,3 | 37°C | 2 weeks | y | n |
| FAV 7 3 | n | Fish scale | *M.luteus* | 5 | y | TRIS pH 9,3 | 37°C | 2 weeks | y | y |
| FAV 8 1 | n | Fish scale | *M.luteus* | 5 | y | TRIS pH 7,5 | 37°C | 1 week | n | n |
| FAV 8 2 | n | Fish scale | *M.luteus* | 5 | y | TRIS pH 9 | 37°C | 1 week | 1 week | 1 week |
| FAV 8 3 | n | Fish scale | *M.luteus* | 5 | y | TRIS pH 9,5 | 37°C | 1 week | 1 week | 1 week |
| FAV 8 4 | n | Fish scale | *M.luteus* | 5 | y | TRIS pH 10 | 37°C | 1 week | 1 week | 1 week |
| FAV 8 5 | n | Fish scale | *M.luteus* | 5 | y | TRIS pH 10,5 | 37°C | 1 week | 1 week | 1 week |
| FAV 8 6 | n | Fish scale | *M.luteus* | 5 | y | TRIS pH 9,5 | 37°C | 1 week | 1 week | 1 week |
| Tissue test ex 1. | n | n | Muscle tissue | 5 | n | seawater | 37°C | 1 week | n | n |
| Tissue test ex 2 | y | NPP | Muscle tissue | 5 | n | seawater | 37°C | 1 week | n | n |
